# Is Shape of a Fresh and Dried Leaf the Same?

**DOI:** 10.1371/journal.pone.0153071

**Published:** 2016-04-05

**Authors:** Dominik Tomaszewski, Angelika Górzkowska

**Affiliations:** 1 Institute of Dendrology of the Polish Academy of Sciences, Parkowa 5, PL-62-035, Kórnik, Poland; 2 Poznań University of Life Sciences, Faculty of Forestry, Wojska Polskiego 71c, PL-60-625, Poznań, Poland; University of Florida, UNITED STATES

## Abstract

Plants kept as dried herbarium specimens share many characteristics with their living counterparts, but there are some substantial differences between them. Due to dehydration, leaves of herbarium specimens change not only their mass and colour, but in many cases change their dimensions, too. The present study aimed to determine whether leaf shape changes during the drying process. A total of 794 pairs of fresh and dried leaves or leaflets of 22 plant taxa were studied. The shape of the blades was quantified using elliptic Fourier analysis combined with principal component analysis. In addition, area and mass of the leaves were measured. Statistical tests were applied for comparing fresh and dried leaves. The results indicate that the preservation process of pressing and drying plants for herbarium purposes causes changes in leaf shape. In general, the shape changes were directional. As the shape of fresh and dried plants is different, it is strongly recommended that shape analyses should be performed on datasets containing either of the leaf types.

## Introduction

Plant science is facing a global shortage of well-trained botanists who can meet demand for accurate species identification, especially in biodiversity research or wildlife conservation, particularly in the tropics. There is an increasing need for quick, efficient and accurate automated identification of plants for scientific or educational purposes (e.g., identification of hazardous plants). One of the solutions to this problem seems to be the use of a laptop, smartphone, or tablet aided system of pattern recognition combined with digital images of plants. There have been many attempts of applying digital morphometrics to solve this problem (e.g., [1–11], but it is still difficult to determine what method performs best. There is a need for an appropriate leaf shape assessment, because software that relies on object shape or boundary, no matter how the system works, needs to be sourced with accurate data to produce reliable results, otherwise an undesired output may be expected (the so called ‘garbage in, garbage out’). Some solutions are based on images of living plants, including those collected on social networks [12], but in our opinion, digitised herbarium specimens may play a very important role in automating species identification [3,7].

Herbaria may serve as near-inexhaustible resources of plant data, including specimen images [13,14]., Several hundred million specimens have been deposited in public herbaria, which contain the widest range of plants, from all continents and environments. Moreover, most species described to date have their representations as type specimens in at least one herbarium in the world. Nowadays, most institutions digitise their collections, so access to a vast number of high-quality images is available.

Plants kept as herbarium specimens share the majority of their characteristics with living plants, but there are some substantial differences between them. Plant fragments or even entire individuals have to be subjected to a preservation treatment that includes pressing and drying. Water removal is essential, as water presence encourages, or at least enables, fast degradation or damage to plant material by bacteria, fungi or insects. Probably colour alterations, especially in flowers, are the most obvious differences between fresh and desiccated plants. Nonetheless, pressing and drying is the easiest and the cheapest method of preserving plant material, and therefore it has been a very common practice used for centuries and still is the universal way of conserving plants in scientific herbaria. This method is ideal in plants due to the flatness of the most prominent organs (e.g., the leaves). During pressing and drying, leaves change relatively less than, for example, flowers, normally having a much more pronounced three-dimensional structure.

Due to dehydration, leaves of herbarium specimens change not only their mass and colours, but in many cases also their dimensions. Recently, more has been discovered about leaf dimensions or area alterations caused by pressing and drying [15–17]. Yet, still very little is known about change in shape. Intuitively, one may assume that although a leaf shrinks, this occurs uniformly throughout the whole leaf; thus, its shape does not change. However, a leaf is not a homogenous structure. It contains tissues or elements of different characteristics: epidermis, mesophyll and veins. Specifically, the epidermis and veins play an important role in strengthening the structure of a leaf. Consequently, changes in blade shape during drying and pressing are clearly possible. There are several methods of quantifying shape of an object. Traditional morphometric methods can be a source of data on leaf shape, including length, width, perimeter and sets of ratios between these basic measurements. This data can be very useful, but often they are too coarse for a detailed quantification of subtleties in shape description. Another option is to use modern morphometrics. This includes methods that analyse the position of so-called landmarks or outlines (boundaries of the objects) [18,19]. We would like to emphasise that shape is considered here as ‘the geometric property of an object invariant under rotation, scale, or translation’ [19]. Thus, the size effect (e.g., area) is filtered out from the leaves and analysed separately.

The present study aimed to determine whether there exist significant differences in the shape of leaf blades between fresh leaves and the same leaves after pressing and drying, or, in other words, whether leaf shape changes during drying for the purpose of making herbarium specimens. If changes occur, it is informative to describe them and to assess their relationships with other selected leaf traits. If a strong relationship were detected, this knowledge would allow simple prediction of the type and degree of shape changes in other plants.

## Materials and Methods

A total of 22 plant taxa from 10 families were studied (Table 1). We selected both woody (deciduous and evergreen) and herbaceous plants, with simple or compound leaves to include a wide range of representatives across flowering plants. At the same time, we opted to select plants with leaf (or leaflet) shapes as simple as possible: flat (not undulate), without lobes or very distinct teeth, and without clear asymmetry. In the case of plants with pinnately-compound leaves, only the terminal leaflet was studied, and in palmately-compound leaves we selected only one of the biggest leaflets.

**Table 1:** Plant material used in the study and significance of differences between groups of fresh and dried leaves in shape parameters, as well as their area loss and mass loss.

Taxon (family)	N	Growth form; leaf type and other remarks	PC1	PC2	Area loss [cm^2^]	Area loss [%]	Mass loss [g]	Mass loss [%]
All samples	794		*		1.3*	7.6*	0.230*	69.4*
*Betula pendula* Roth (Betulaceae)	36	woody (tree), deciduous	*	*	1.0*	8.2*	0.101*	60.2*
*Fagus sylvatica* L. (Fabaceae)	34	woody (tree), deciduous	*		1.8*	3.5*	0.333*	73.8*
*Ficus retusa* L. (Moraceae)	36	woody (tree), evergreen	*	*	2.9*	15.2*	0.386*	73.1*
*Fraxinus ornus* L. (Oleaceae)	29	woody (tree), deciduous; pinnately-compound leaves	*	*	0.8*	4.9*	0.179*	68.4*
*Lamium album* L. (Lamiaceae)	35	herbaceous	*	*	0.5*	5.6*	0.103*	73.5*
*Lupinus polyphyllus* Lindl. (Fabaceae)	37	herbaceous; palmately-compound leaves	*	*	0.7*	5.6*	0.238*	82.8*
*Oemleria cerasiformis* (Torr. & A.Gray) J.W. Landon (Rosaceae)	32	woody (shrub), deciduous	*		1.3*	4.9*	0.238*	68.9*
*Plantago lanceolata* L. (Plantaginaceae)	29	herbaceous	*	*	2.3*	8.6*	0.606*	86.3*
*Plantago major* L. (Plantaginaceae)	28	herbaceous	*		2.9*	8.8*	0.606*	78.6*
*Robinia pseudoacacia* L. (Fabaceae)	31	woody (tree), deciduous; pinnately-compound leaves	*	*	0.7*	9.5*	0.058*	65.7*
*Rosa arvensis* Huds. (Rosaceae)	29	woody (shrub), deciduous; pinnately-compound leaves	*		0.3*	7.4*	0.018*	51.9*
*Rosa arvensis* Huds. (Rosaceae)	33	woody (shrub), deciduous; pinnately-compound leaves	*	*	0.4*	8.9*	0.032*	52.0*
*Salix pentandra* L. (Salicaceae)	28	woody (shrub), deciduous	*		1.8*	7.0*	0.299*	66.2*
*Secale cereale* L. (Poaceae)	30	herbaceous	*	*	0.7*	12.1*	0.065*	72.0*
*Sorbus aucuparia* L. (Rosaceae)	34	woody (tree), deciduous; pinnately-compound leaves	*	*	0.4*	11.4*	0.034*	59.4*
*Syringa ×chinensis* Willd. (Oleaceae)	38	woody (shrub), deciduous	*	*	0.6*	5.6*	0.117*	63.9*
*Syringa ×prestoniae* McKelvey ‘Esterka’ (Oleaceae)	37	woody (shrub), deciduous	*	*	4.6*	9.7*	0.787*	75.8*
*Syringa josikaea* J. Jacq. (Oleaceae)	30	woody (shrub), deciduous	*	*	2.5*	5.7*	0.500*	69.6*
*Syringa meyeri* C.K. Schneid. (Oleaceae)	35	woody (shrub), deciduous	*	*	0.9*	11.3*	0.100*	64.1*
*Syringa vulgaris* L. ‘Princesse Clementine’ (Oleaceae)	32	woody (shrub), deciduous		*	2.4*	6.0*	0.523*	66.0*
*Trifolium repens* L. (Fabaceae)	36	herbaceous; pinnately-compound leaves	*		0.1*	7.5*	0.012*	73.7*
*Vinca minor* L. (Apocynaceae)	31	subshrub, evergreen; previous year leaves	*		0.2*	4.0*	0.060*	77.4*
*Vinca minor* L. (Apocynaceae)	39	subshrub, evergreen; current year leaves	*		0.2*	3.9*	0.081*	64.2*
*Wisteria floribunda* (Willd.) DC. (Fabaceae)	35	woody (liana), deciduous; pinnately-compound leaves	*	*	1.5*	8.1*	0.150*	76.3*

N = number of leaves or leaflets used in the analysis

traits marked with asterisk * are significant with p < 0.05

Two additional aspects were investigated. The first is a comparison between leaves from shady and sunny places. For this, leaves of *Rosa arvensis* were sampled from plants that grew in such places. The second aspect is a comparison between young and old leaves from an evergreen species. For this, current-year and previous-year leaves from *Vinca minor* were collected and analysed.

The leaves were sampled between May and July. Only healthy, undamaged leaves without any signs of herbivory, discolorations, developmental irregularities or abnormalities were selected, preferably from a single individual. All plants grew in the Kórnik Arboretum (Poland) belonging to the Institute of Dendrology of the Polish Academy of Sciences, or growing in its proximity.

All leaves from a taxon were collected on one day and immediately transported to the laboratory where they were processed within a couple of hours. To reduce evaporation, they were kept in plastic bags in a refrigerator (+4°C) awaiting processing. Petioles or petiolules were cut off. Each leaf was weighted with an analytical balance (WPS 180/C/2, Radwag, Poland) and marked with a unique number. The leaves were then scanned with an Epson Perfection V700 scanner (24-bit RGB, 300 dpi).

Weighted and scanned leaves were placed in a herbarium press. The press consisted of two wooden plates (305 × 445 mm) with holes (Ø 25 mm). Fresh plant material was put between paper sheets and interleaved with blotting paper. This pile was then placed between the plates. The upper plate was pressed against the pile and the bottom plate with a roller and ropes. The force used for pressing was manually adjusted depending on the amount and kind of plant material. The plants were dried in the press for 5–7 days at ambient temperature (18–25°C) and humidity (40–55%). To make the drying more efficient, the blotting paper sheets were changed when necessary. This is the usual drying procedure in the KOR herbarium.

Dried leaves were reweighted and rescanned the same way as fresh ones. Leaves with even small damage caused by drying and pressing were eliminated from analyses. This way, we obtained a pair of digital images and data on their mass for each lamina. The images are available at: Tomaszewski D. fresh and dried leaf shapes. 2016. RepOD. http://dx.doi.org/10.18150/repod.6957786.

Elliptic Fourier analysis (EFA) is one of the methods used in geometric morphometrics for shape analyses, especially for landmark-poor outlines [18,19]. It is a very powerful and informative method for objective shape quantification. It uses a Fourier transformation of an outline to obtain a set of quantitative variables, called harmonics, each described by four coefficients [20]. The coefficients may be subjected to multivariate statistical methods, mainly principal component analysis (PCA), which is a statistical method that reduces the number of variables (dimensions) without much loss of information. Moreover, EFA with PCA allows visualisation and reconstruction of shapes via inverse Fourier transformation, which is very helpful when the biological significance of the shape is considered.

Since leaves are simple two-dimensional objects, researchers began to use EFA as soon as computer techniques of image acquisition and processing became available [21–33]. For this reason, we decided to perform an analysis of potential shape changes by applying EFA.

The next step of the study consisted in EFA of the digital images with SHAPE v. 1.3 [34]. The program provided the normalised elliptic Fourier descriptors (EFDs) and leaf area for images of both fresh and dried leaves.

In our study, four coefficients (a, b, c and d) for 20 harmonics were calculated by the Fourier transformation of a chain-coded contour [20] with the use of the normalisation method based on the farthest point on the contour from its centroid (the longest radius). This way, coefficients were normalised to be invariant with respect to size, rotation and starting point. The program also generated the data on the blade area of each leaf.

The EFDs precisely quantify leaf shape, and they can be analysed with multivariate statistical methods, mainly PCA. In our study, a PCA on covariances was performed on symmetric and asymmetric coefficients (a and d, as well as b and c, respectively) for all data (pairs of fresh and dried leaf blades). This way, 80 variables that describe leaf shape were reduced to only two main principal components (PCs) and used for further analyses.

The following traits were measured or calculated for both fresh and dried blades: area, mass, PC1 and PC2. In this regard, the samples can be treated as dependent. For determining whether leaves differed significantly before and after pressing and drying, paired difference tests were implemented. For testing normality of distributions, the Shapiro–Wilk test was used. Depending on the test results, a paired Student’s t-test or its non-parametric alternative, the Wilcoxon signed-rank test, was performed. Based on the mass, area and shape measurements, additional traits were calculated.

Each pair of blades (fresh and dried) has its representation in the PC1–PC2 space as a pair of points. The length of the vector, calculated as the Euclidean distance between the two points (i.e., sqr(∆PC1^2^ + ∆PC2^2^), indicates the magnitude of shape displacement: the longer the vector, the bigger the shape change. Similarly, angle α between the vector and the PC1-axis (S1 Fig) is related to direction of shape change. We can use sin α or cos α as measures of directionality along the PC1 or PC2 axes, respectively. Sin α is calculated as the ratio between the opposite side to the angle α (∆PC1 = PC1 (fresh)—PC1 (dried)) and hypotenuse (i.e., the length of the corresponding vector). This means that the more pointed the vector toward lower values of PC1 (i.e., α → 90°), the bigger the sin α values (the relationship is not linear). Likewise, cos α is calculated as the ratio between the adjacent side to the angle α (∆PC2 = PC2 (fresh)—PC2 (dried)) and hypotenuse (i.e., the length of the vector). In this case, the more pointed the vector toward lower values of PC2 (i.e., α → 0°), the bigger the cosine values.

PC1 and PC2 were measured or calculated for both fresh and dried blades and treated as dependent variables. A paired Student’s t-test or its non-parametric alternative, the Wilcoxon signed-rank test, was applied for assessing the differences between fresh and dried leaves.

Statistical analyses were performed with Statistica 9 (StatSoft Polska) and JMP 11 (SAS Institute Inc.). Package ggplot2 for R was used for some diagrams. Detailed results on basic statistics, as well as on the tests and correlations, are found in S1–S8 Tables.

## Results

Seven hundred and ninety-four pairs of fresh and dried leaf blades were analysed. For shape data, PCA on covariances was performed on the set. No data on mass or leaf area were used in this step. The first two PCs (PC1 and PC2) accounted for 80.4% and 11.4% of variance, respectively (in total 91.8%). The others explained significantly much less variance (approximately one order of magnitude); thus, they were excluded, and for further analyses only PC1 and PC2 were used.

**Fig 1:**
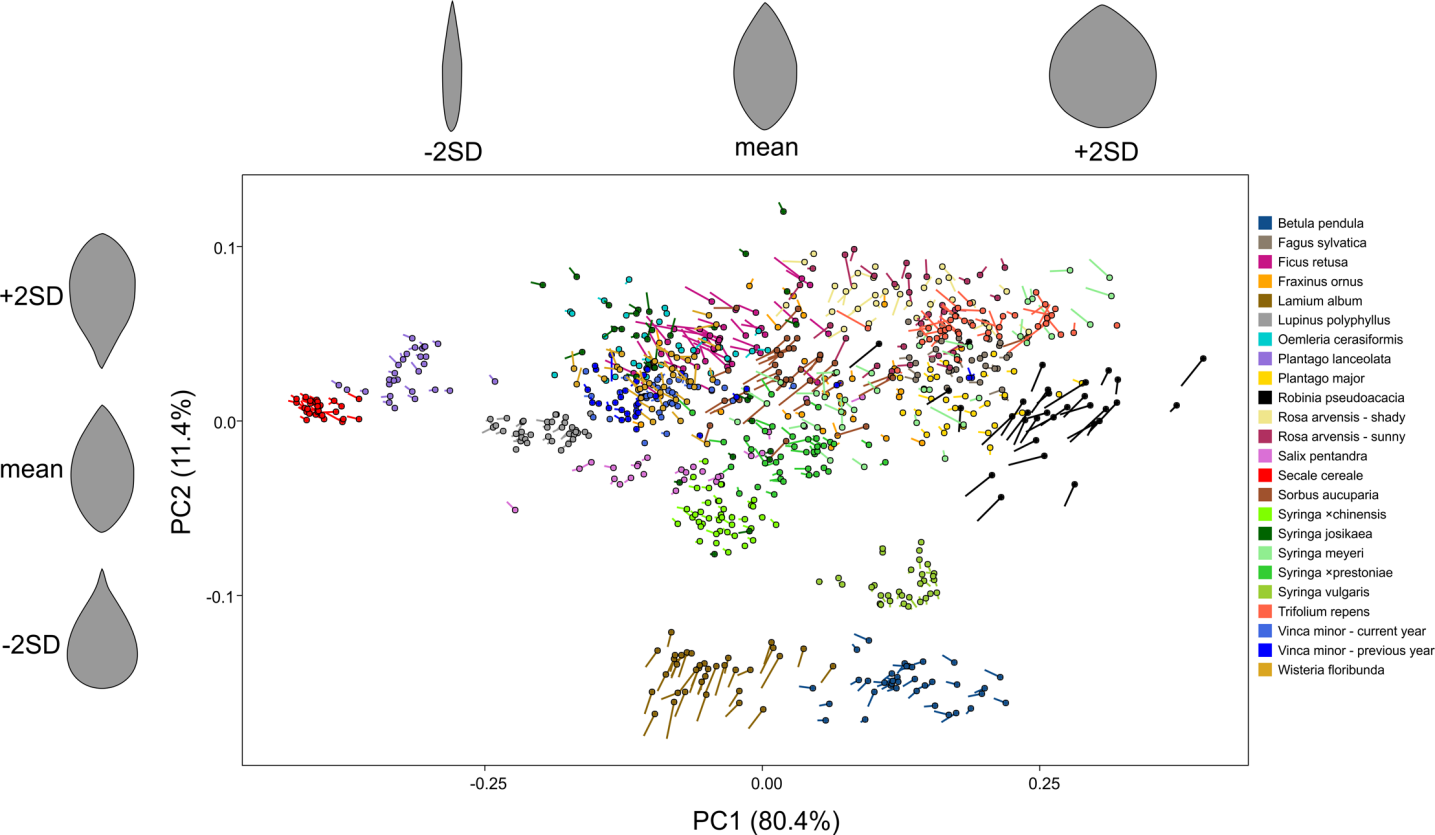
Scatterplot for results of principal component analysis of elliptic Fourier descriptors. Small circles indicate position of fresh leaf shape in the PC1–PC2 morphospace, while the lines show the shape change. Individual scatterplots are presented in Fig 2. Patterns of variation along PC1 and PC2 axes are shown on the top and on the left of the plot, by a mean shape and shapes +2 and -2 standard deviation (SD) distant from the mean.

**Fig 2:**
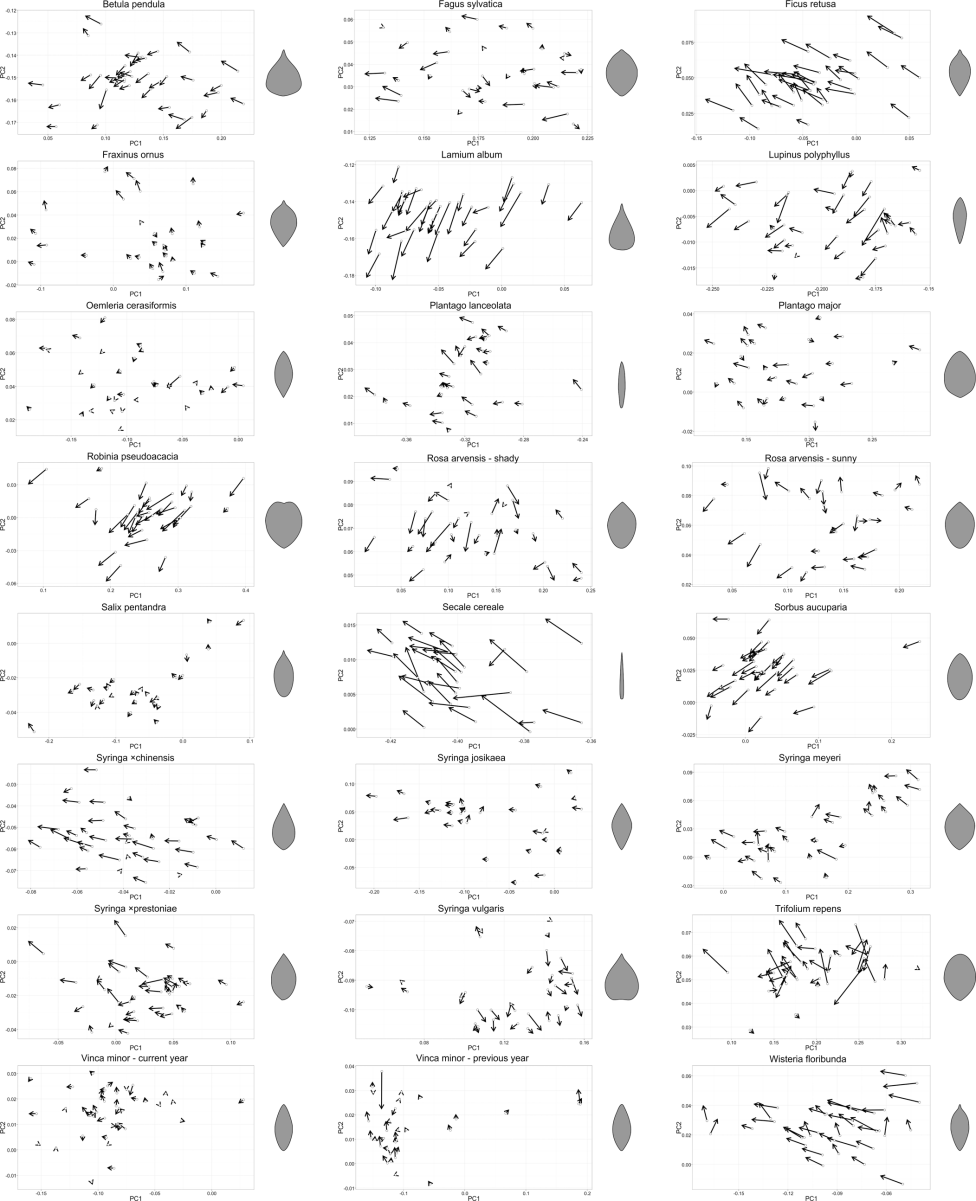
Shape changes resulted from principal component (PC) analysis of elliptic Fourier descriptors for fresh and dried leaves for each group of analysed plants. Arrows represent shape changes in the PC1–PC2 space. The starting point indicates shape of a fresh leaf and its head corresponds to its shape after drying. Vector lengths are, in general, not comparable between diagrams, as their scaling is not identical. Leaf silhouettes on the right present mean shapes of fresh leaves.

A summary scatterplot (Fig 1) presents results of the PCA. Points indicate the position of fresh leaf shape in the two-dimensional, PC1–PC2 space, while changes in shape are presented as lines. The scatterplot is typical for the great diversity of shapes analysed here. Blades group according to their shape. Many species (such as *Secale cereale*, *Syringa ×chinensis*, *Lupinus polyphyllus*, *Plantago lanceolata* and *Syringa vulgaris*) are clearly separated in this plot because their leaf shapes are well-defined and the observed variability is limited (see S3 and S4 Tables with additional data that resulted from PCA). In contrast, leaf shapes are much more diverse in some other species, e.g., *Syringa meyeri*. Individual plots for each plant group are presented in Fig 2.

**Fig 3:**
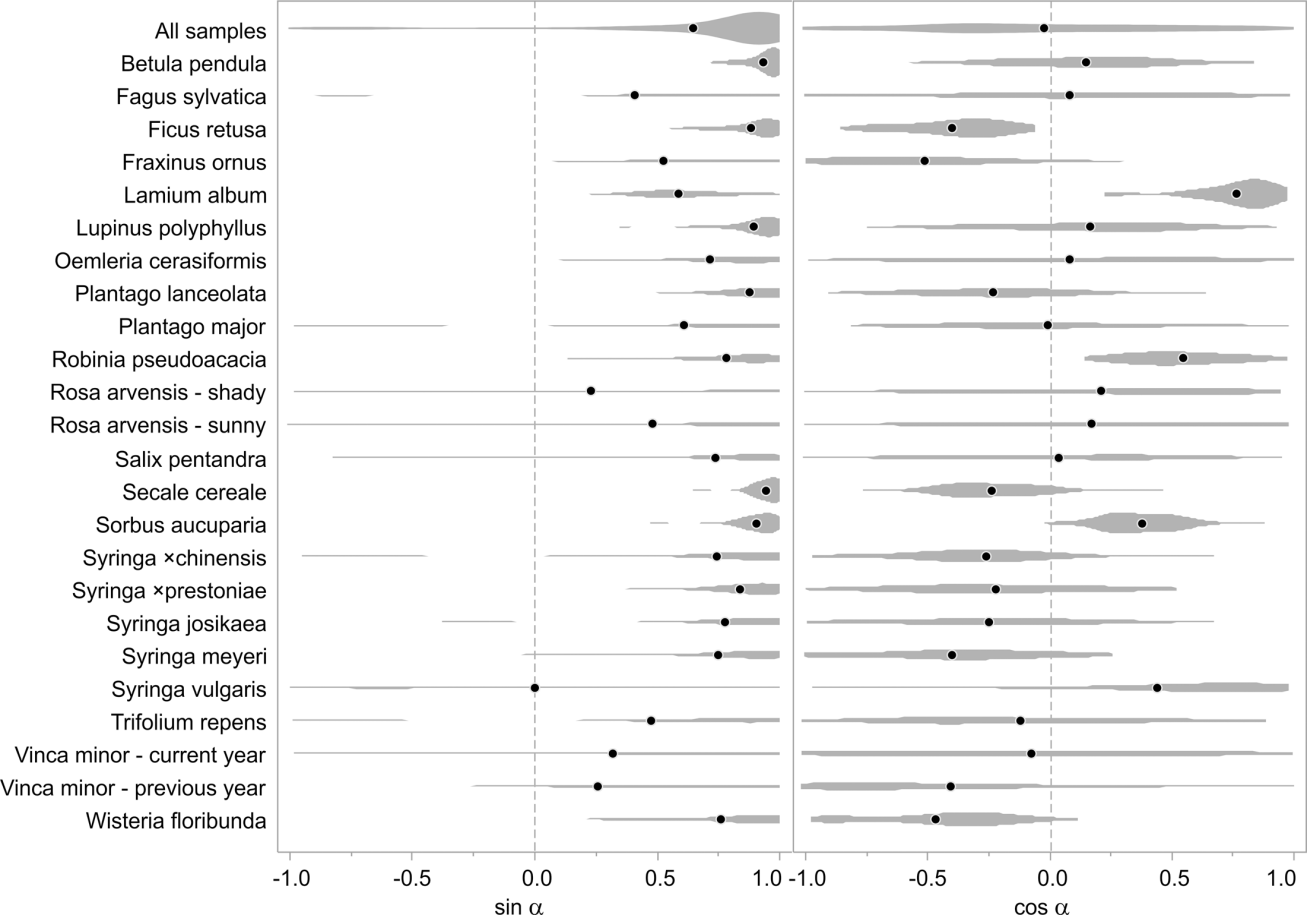
Values distribution of sine and cosine of the angles between shape-change vectors and the principal component 1 (PC1)-axis. Mean values marked with black points. The sin α values correspond to direction of the vector along PC1-axis, while cos α values correspond to its direction along the PC2-axis. All sin α means are positive, which points out that there exists a strong generalised tendency of directional shape change toward lower values of PC1. No general trend has been detected in cos α values, although strong tendencies can be observed for some species.

The distribution of sin α and cos α values is presented in Fig 3. Statistics of vector length and sin α and cos α are presented in the Supporting Information (S5 and S6 Tables). It is noteworthy that all sin α means (except for *Syringa vulgaris*) are positive and that the values tend to be distributed around 1 (thus, α → 90°). No general trend has been detected in cos α values, although strong tendencies can be seen in some species (such as *Ficus retusa*, *Lamium album*, *Robinia pseudoacacia*, *Secale cereale* and *Sorbus aucuparia*).

Regarding PC1, only fresh and dried leaves of *Syringa vulgaris* do not seem to differ significantly. In terms of PC2, the differences for *Fagus sylvatica*, *Oemleria cerasiformis*, *Plantago major*, *Rosa arvensis* (sunny), *Salix pentandra*, *Trifolium repens*, and *Vinca minor* are not significant (Table 1).

Differences in shape between fresh and dried leaves, expressed in at least one PC, are significant in all groups and, interestingly, shape changes are generally directional, i.e., mainly occurring along the PC1 axis toward lower values (Figs 2 and 3), which can be translated into conventional descriptive terms as ‘becoming narrower’. Shifts toward lower or higher values of PC2 are not well defined; however, strong tendencies can be observed for some species (e.g., *Lamium album*). In those species, leaf blades do not become narrower evenly along their long axis. In *Lamium album* the area closer to the tip shrinks relatively more than the area closer to its base, while in *Ficus retusa*, the opposite is true (Figs 2 and 3).

For all leaves, area and mass were measured before and after drying and pressing (S1 and S2 Tables) and were treated as dependent variables. A paired Student’s t-test or Wilcoxon signed-rank test was applied for assessing the differences between fresh and dried blades. In our study, the leaves lost 52–86% of their mass (mean 69%), and their area decreased between 3.5% and 15.2% (mean 7.6%). The result of the comparison clearly indicates that both area and mass of the leaves in each group, as well as leaves pooled together, decreased significantly (Table 1, S7 Table).

In the comparison of leaflets from sunlit and shade leaves of *Rosa arvensis* we did not detect any major differences in terms of basic shape changes (∆PC1, ∆PC2, vector length, sin α). The same applies to previous- and current-year leaves of *Vinca minor*. The only distinct dissimilarity consisted in their water content (higher in current-year leaves) and thus specific leaf area (higher in previous-year leaves).

**Fig 4:**
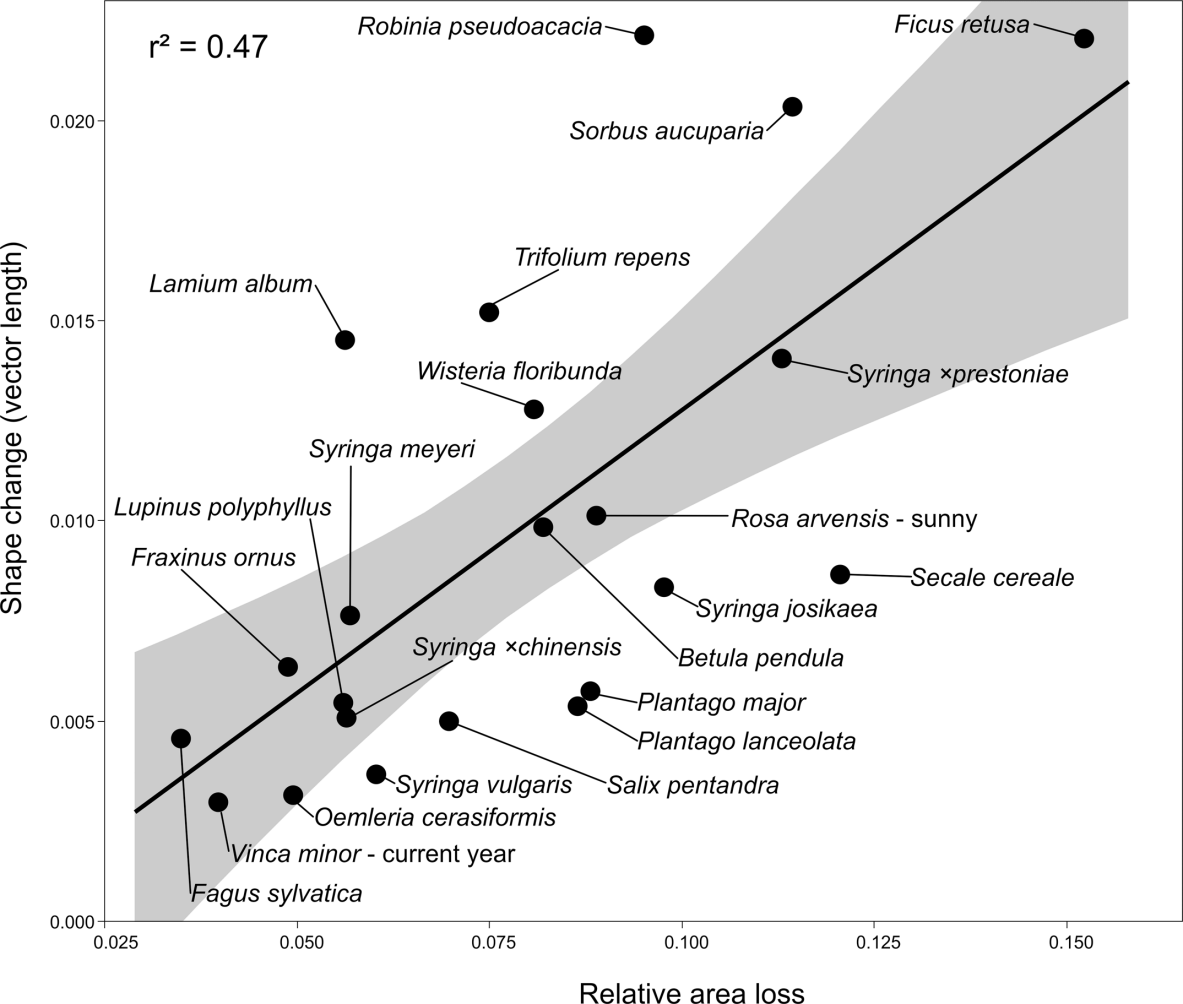
Relationship between relative area loss and shape change expressed as vector length. Mean values for species were used, with 95% confidence of fit.

Preliminary correlation analyses between shape parameters and other measurements (i.e., mass and area of fresh leaves, mass and area of dried leaves, specific leaf area (SLA, calculated as the ratio of fresh leaf area and dried leaf mass), mass and area loss, and relative area and mass loss) were performed due to the lack of normal distribution in some groups and differences in numbers of observations. These provisional results show that the strongest relationships exist between relative area loss and shape change (expressed as vector length; S8 Table). The maximum values of r coefficients were detected for *Secale cereale*, *Lupinus polyphullus* and *Plantago lanceolata* (r = 0.86, 0.82 and 0.80, respectively, p < 0.001). When mean values for each species (previous-year leaves of *Vinca minor* and *Rosa arvensis* from shade excluded) were used (Fig 4), the r coefficient was still high (0.69, thus r^2^ = 0.47; p = 0.004). Additionally, for assessing differences in relative area loss between woody and herbaceous taxa, a Wilcoxon test was applied. However, the differences are not significant (with p < 0.05).

## Discussion

Leaf shape is very important not only in species identification, but also in other studies, such as plant ecology and physiology. Both fresh and dried leaves may be used, but it should be clear whether the leaf shape in a living plant is identical to the leaf shape in a herbarium specimen. If they are not identical, and both fresh and dried leaves are used, an error could occur leading to the wrong conclusions. For this reason, the present study aimed to determine whether there exist differences in shape between leaves before and after going through the pressing and drying process. We approached this question by applying EFA that analyses the objects represented by their outline. To the best of our knowledge, no such study has been carried out using similar morphometric and statistical tools. Our results show that leaf shape changes significantly during the herbarium drying process in all plants we studied (Table 1).

Leaves are diverse in their anatomy and morphology and variation exists even between closely related species. However, at the same time, leaves have many traits in common. The results we obtained in the study can be interpreted as an expression of this dualism.

Veins, hypodermis, sclerenchymatous ribs, bundle sheath extensions and hydrostatic pressure provide support for leaf structure [35]. In our opinion, the taxa analysed here share a widespread pattern of major changes because their leaves have a similar basic vascular skeleton. Our results indicate that the primary vein framework, both pinnate and palmate, strengthens the blade along its long axis, so that changes in shape consist mainly of becoming narrower. This is congruent with results by Jeong et al. [36] who simulated morphology changes in drying leaves and modelled the wrinkles caused by non-homogeneous shrinkage due to dehydration. One of their conclusions was that the regions away from the main vein wrinkle faster and to a greater extent. The observation that the length-to-width ratio becomes larger after drying is also in accordance with results obtained by Parnell et al. [16] and Wooley [37]. In the latter study, pieces of maize leaves did not change measurably in length, while their width diminished by 2% (the leaf fragments were only 28 × 32 mm, therefore, they likely did shrink to some extent in both directions, but much more significantly in width). In summary, our observations also confirm that leaves shrink more in width than in length.

While major changes in shape (c. 80% of variance) consist of leaves becoming narrower, minor changes (c. 11%) are not that strong and evident. These are species-specific and may be explained by diversity of leaf anatomy, especially of the secondary venation system, mesophyll and epidermis thickness, lignin (hypothesised by Juneau & Tarasoff [15]) and cellulose or water content. This is not surprising considering that a leaf does not have a homogenous structure. Structure varies between species, and leaves do not shrink equally in all directions.

Different leaf morphologies affect the type of shape changes during the drying process. For example, we predict that shape changes of leaf lobes and teeth may follow the same trend we observed in simple leaves with entire margins, i.e., they may become narrower. However, these changes are harder to quantify and how they influence the overall change in leaf shape is difficult to predict and requires additional studies.

Plants contain considerable amounts of water–c. 70% [38]–that has to be removed when making herbarium specimens. At the same time, plant cells have cell walls, which are not rigid. It is obvious that desiccation changes both the cell volume and mass and structure of cell walls. As a result, cell mass decreases dramatically and cells shrink, triggering a reduction in the whole leaf area.

In our study, leaves lost 52–86% of their mass, and their area decreased by 3.5–15.2%. Irrespective of the degree of reduction, in all species analysed, these changes were significant (Table 1). Unlike results from studies by Juneau & Tarasoff [15] and Blonder et al. [39], the values of leaf area decrease we obtained are much lower (S1 Table). On average, pressed leaves in those studies shrunk by c. 18% and c. 22%, respectively. In turn, Queenborough & Porras [17] reported that leaves used in their experiment shrunk by only 8% on average. These results are quite unexpected. It seems possible that the differences in leaf area decrease between studies might stem from an overrepresentation of woody species, which, in general, are characterised by less significant shrinkage. Both Juneau & Tarasoff [15] and Blonder et al. [39] found that leaves of woody species shrink significantly less than the others. However, in our study, no relationship between growth form and shrinkage was detected, with herbaceous taxa being more or less randomly dispersed among woody species (tested by comparing means for woody and herbaceous taxa by the Wilcoxon test). Alternatively, different methods of pressing may be a reason for such differences. Supposedly, if the leaves are pressed more firmly, this may prevent shrinkage to some degree.

We think that the changes are correlated with type of leaves, their anatomical structure, or age. This may also include methods of preserving plant material. The drying method we used is standard for temperate collections. Specimens collected in the tropics may be treated in ethanol before drying. From the literature [16], we know that this method results in greater shrinkage than conventional drying. In the meantime, we can hypothesise that this ‘greater shrinkage’ may be correlated with shape changes. We believe that our preliminary study may inspire others to investigate such remaining questions.

Queenborough & Porras [17] associated the degree of shrinkage with softness of leaves and life history strategies in different families. In contrast, Blonder et al. [39] considered that ‘evolutionary history is not especially helpful for predicting shrinkage’. Both studies used large sets of species (123 and 175, respectively). Base on our results, we conclude that leaf area decrease is also very species-specific.

Both leaf mass and leaf area are very important parameters in plant ecology and physiology as they are used for calculation of SLA, LMA (leaf mass per area), stomatal density, and complexity of leaf shape (leaf perimeter-to-area ratio), to mention only the most widely used parameters. If accurate leaf area and/or area-dependent leaf parameters are not used, errors may result [15]. In such cases, it is highly recommended that only fresh leaves be used, and, if not possible, the degree of shrinkage should be known for calculation adjustments [40]. According to Torrez et al. [41], it is also possible to use a special protocol to obtain predicted SLA.

In this study, we attempted to estimate differences in shape, area and mass between fresh and dried leaves from herbarium specimens. Differences exist and are statistically significant. For this reason, special attention should be paid to the choice of leaf material. Our results indicate that for correct shape analyses only leaves from either living plants or dried herbarium specimens should be used. If this is not possible, we think that solutions such as SLA predictions, should be considered. We hope that our results may be a starting point for additional research.

## Supporting Information

S1 Fig Angle α between the vector and PC1-axis. (TIF)

S1 Table Basic statistics on area of analysed leaves/leaflets. SD = standard deviation; SW p–p-value in Shapiro-Wilk test, where ^N^ indicates normal distribution.
(PDF)

S2 Table Basic statistics on mass of analysed leaves/leaflets. SD = standard deviation; SW p = p-value in Shapiro-Wilk test, where ^N^ indicates normal distribution.
(PDF)

S3 Table Basic statistics on principal component 1 (PC1) of analysed leaves/leaflets. SD = standard deviation; SW p = p-value in Shapiro-Wilk test, where ^N^ indicates normal distribution.
(PDF)

S4 Table Basic statistics on principal component 2 (PC2) of analysed leaves/leaflets. SD = standard deviation; SW p = p-value in Shapiro-Wilk test, where ^N^ indicates normal distribution.
(PDF)

S5 Table Basic statistics on sin α and cos α. SD = standard deviation; SW p = p-value in Shapiro-Wilk test, where ^N^ indicates normal distribution.
(PDF)

S6 Table Basic statistics on shape change and specific leaf area (SLA) of analysed leaves/leaflets. SD = standard deviation; SW p = p-value in Shapiro-Wilk test, where ^N^ indicates normal distribution.
(PDF)

S7 Table Results (p-values) of paired Student’s t-test or Wilcoxon signed-rank test for repeated measurements of principal component 1 (PC1), PC2, area and mass of analysed fresh and dried leaves/leaflets. Bolded values (p > 0.05) indicate that the compared populations do not differ significantly.
(PDF)

S8 Table Correlations between shape change (vector length) and selected variables expressed as coefficient r values. Bolded r values are significant with p < 0.05.
(PDF)
