# Supplementary figures and images for: Is Shape of a Fresh and Dried Leaf the Same?
Source: PLoS One. 2016 Apr 5;11(4):e0153071. doi: 10.1371/journal.pone.0153071 (PMC4821626; doi:10.1371/journal.pone.0153071)

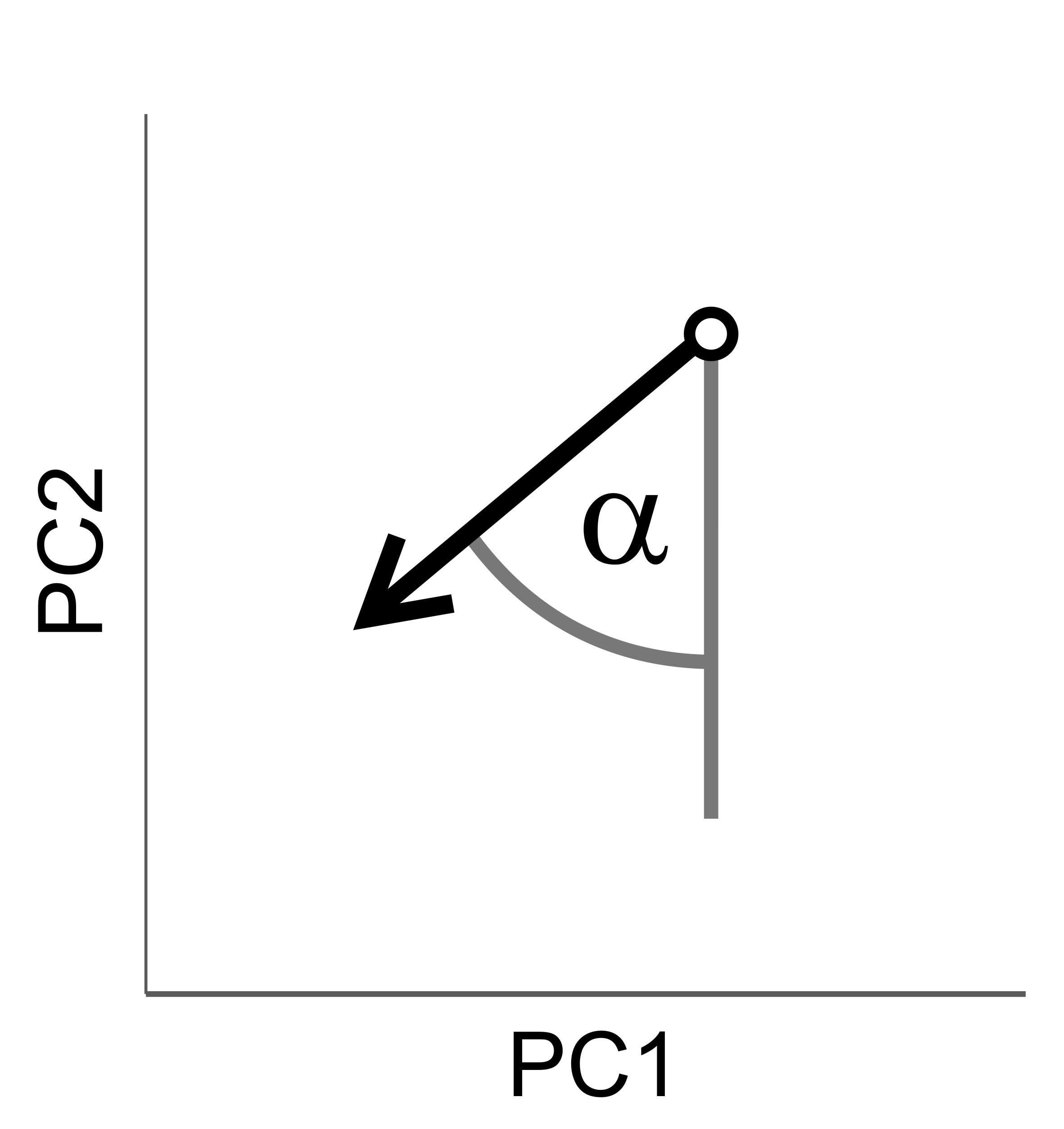

Supplement: S1 Fig — (TIF) [file pone.0153071.s001.tif]
