# Supplementary material for: Is Shape of a Fresh and Dried Leaf the Same?
Source: PLoS One. 2016 Apr 5;11(4):e0153071. doi: 10.1371/journal.pone.0153071 (PMC4821626; doi:10.1371/journal.pone.0153071)
Supplement: S1 Table — SD = standard deviation; SW p–p-value in Shapiro-Wilk test, where N indicates normal distribution. (PDF) [file pone.0153071.s002.pdf]

Table S1. Basic statistics on area of analysed leaves/leaflets (SD = standard deviation; SW p – p-value in Shapiro-Wilk test, where ^{N} indicates normal distribution).

		**Area (fresh) [cm^2]**					**Area (dried) [cm^2]**					**Area loss [cm^2]**					**Area loss [%]**				
**Group**	**N**	**Mean**	**Min**	**Max**	**SD**	**SW p**	**Mean**	**Min**	**Max**	**SD**	**SW p**	**Mean**	**Min**	**Max**	**SD**	**SW p**	**Mean**	**Min**	**Max**	**SD**	**SW p**
All samples	794	17.9	0.7	83.2	16.4	0.00	16.6	0.6	81.7	15.4	0.00	1.3	0.1	8.1	1.3	0.00	7.6	1.8	21.1	3.3	0.00
Betula pendula	36	11.6	6.8	18.2	3.2	0.19^{N}	10.7	6.2	16.7	2.9	0.23^{N}	1.0	0.4	1.7	0.3	0.85^{N}	8.2	5.4	9.7	0.8	0.01
Fagus sylvatica	34	51.3	28.1	83.2	12.0	0.63^{N}	49.5	26.9	81.7	11.7	0.49^{N}	1.8	0.7	3.2	0.7	0.11^{N}	3.5	1.8	5.4	1.1	0.10^{N}
Ficus retusa	36	19.2	13.3	29.4	4.1	0.18^{N}	16.3	11.2	25.8	3.6	0.17^{N}	2.9	1.9	4.3	0.6	0.08^{N}	15.2	11.7	17.8	1.5	0.26^{N}
Fraxinus ornus	29	16.2	5.8	27.5	4.4	0.66^{N}	15.5	5.5	26.6	4.3	0.67^{N}	0.8	0.3	1.1	0.2	0.08^{N}	4.9	3.2	6.9	0.9	0.47^{N}
Lamium album	35	9.7	5.9	19.6	2.6	0.00	9.2	5.2	18.5	2.5	0.00	0.5	0.3	1.1	0.2	0.01	5.6	2.9	10.9	1.6	0.00
Lupinus polyphyllus	37	13.0	6.5	17.6	3.2	0.04	12.3	6.0	16.8	3.2	0.03	0.7	0.3	1.1	0.2	0.28^{N}	5.6	2.3	10.6	1.7	0.19^{N}
Oemleria cerasiformis	32	27.7	17.4	42.2	6.3	0.38^{N}	26.4	16.2	40.7	6.1	0.38^{N}	1.3	0.8	2.3	0.4	0.37^{N}	4.9	3.2	7.5	1.1	0.27^{N}
Plantago lanceolata	29	26.0	3.8	48.1	9.2	0.14^{N}	23.7	3.5	44.1	8.3	0.20^{N}	2.3	0.3	5.0	1.2	0.03	8.6	3.8	15.2	2.5	0.57^{N}
Plantago major	28	33.3	16.1	55.6	10.4	0.34^{N}	30.4	14.3	50.2	9.6	0.35^{N}	2.9	1.7	5.4	0.9	0.07^{N}	8.8	6.6	10.8	1.1	0.43^{N}
Robinia pseudoacacia	31	7.3	3.7	11.3	2.2	0.39^{N}	6.6	3.4	10.5	2.0	0.35^{N}	0.7	0.3	1.1	0.2	0.40^{N}	9.5	6.6	13.9	1.9	0.40^{N}
Rosa arvensis - shady	33	3.5	2.3	6.2	0.9	0.03	3.3	2.1	5.7	0.8	0.04	0.3	0.1	0.5	0.1	0.18^{N}	7.4	4.3	9.9	1.1	0.87^{N}
Rosa arvensis - sunny	29	4.2	2.3	5.9	1.1	0.21^{N}	3.8	2.0	5.4	1.0	0.16^{N}	0.4	0.3	0.5	0.1	0.29^{N}	8.9	6.8	12.9	1.6	0.01
Salix pentandra	28	25.8	10.0	46.0	10.5	0.31^{N}	24.1	9.1	42.4	9.8	0.32^{N}	1.8	0.8	3.6	0.8	0.06^{N}	7.0	5.2	9.6	1.1	0.16^{N}
Secale cereale	30	6.3	2.5	16.2	3.1	0.01	5.6	2.0	14.4	2.8	0.01	0.7	0.2	1.8	0.4	0.06^{N}	12.1	5.8	21.1	3.5	0.07^{N}
Sorbus aucuparia	34	3.9	2.4	7.1	1.1	0.01	3.5	2.0	6.2	0.9	0.01	0.4	0.2	0.9	0.1	0.01	11.4	7.2	16.1	2.0	0.46^{N}
Syringa ×chinensis	38	10.4	6.1	16.1	3.0	0.04	9.8	5.7	15.0	2.8	0.03	0.6	0.3	1.2	0.2	0.03	5.6	3.7	7.2	0.9	0.65^{N}
Syringa ×prestoniae	37	46.9	23.5	70.5	13.7	0.30^{N}	42.3	21.0	65.8	12.4	0.44^{N}	4.6	1.9	8.1	1.6	0.26^{N}	9.7	6.2	13.6	1.7	0.84^{N}
Syringa josikaea	30	43.0	19.8	80.2	17.5	0.04	40.4	18.7	76.1	16.1	0.06^{N}	2.5	1.0	7.2	1.5	0.00	5.7	3.1	9.4	1.4	0.58^{N}
Syringa meyeri	35	8.2	5.1	12.0	1.7	0.64^{N}	7.3	4.4	10.8	1.5	0.49^{N}	0.9	0.5	1.5	0.2	0.25^{N}	11.3	8.3	15.8	1.7	0.08^{N}
Syringa vulgaris	32	40.4	28.2	55.6	7.2	0.62^{N}	38.0	26.1	52.6	6.9	0.67^{N}	2.4	1.4	3.5	0.5	0.15^{N}	6.0	4.3	8.3	1.0	0.75^{N}
Trifolium repens	36	1.4	0.7	2.2	0.4	0.70^{N}	1.3	0.6	2.1	0.4	0.72^{N}	0.1	0.1	0.2	0.0	0.10^{N}	7.5	4.0	14.1	2.2	0.24^{N}
Vinca minor - current year	39	4.0	2.7	6.1	1.0	0.05	3.8	2.5	5.9	0.9	0.06^{N}	0.2	0.1	0.3	0.1	0.00	4.0	2.3	5.8	0.9	0.29^{N}
Vinca minor - previous year	31	4.3	2.7	6.3	0.9	0.43^{N}	4.2	2.6	6.0	0.8	0.40^{N}	0.2	0.1	0.3	0.0	0.55^{N}	3.9	2.6	5.7	0.8	0.50^{N}
Wisteria floribunda	35	18.3	4.8	30.2	6.0	0.47^{N}	16.8	4.5	27.2	5.4	0.47^{N}	1.5	0.3	3.0	0.6	0.21^{N}	8.1	4.6	11.2	1.5	0.68^{N}
