# Supplementary material for: Is Shape of a Fresh and Dried Leaf the Same?
Source: PLoS One. 2016 Apr 5;11(4):e0153071. doi: 10.1371/journal.pone.0153071 (PMC4821626; doi:10.1371/journal.pone.0153071)
Supplement: S2 Table — SD = standard deviation; SW p = p-value in Shapiro-Wilk test, where N indicates normal distribution. (PDF) [file pone.0153071.s003.pdf]

Table S2. Basic statistics on mass of analysed leaves/leaflets (SD = standard deviation; SW p = p-value in Shapiro-Wilk test, where ^{N} indicates normal distribution).

		Mass (fresh) [g]					Mass (dried) [g]					Mass loss [g]					Mass loss [%]				
**Group**	**N**	**Mean**	**Min**	**Max**	**SD**	**SW p**	**Mean**	**Min**	**Max**	**SD**	**SW p**	**Mean**	**Min**	**Max**	**SD**	**SW p**	**Mean**	**Min**	**Max**	**SD**	**SW p**
All samples	794	0.315	0.006	1.924	0.319	0.00	0.085	0.002	0.412	0.079	0.00	0.230	0.004	1.670	0.249	0.00	69.4	44.4	92.6	9.1	0.00
Betula pendula	36	0.167	0.088	0.277	0.050	0.38^{N}	0.067	0.035	0.111	0.020	0.32^{N}	0.101	0.052	0.166	0.030	0.38^{N}	60.2	57.2	64.7	2.0	0.09^{N}
Fagus sylvatica	34	0.452	0.203	0.694	0.111	0.89^{N}	0.119	0.054	0.186	0.031	0.78^{N}	0.333	0.149	0.508	0.081	0.92^{N}	73.8	72.2	75.8	0.9	0.50^{N}
Ficus retusa	36	0.528	0.341	0.784	0.117	0.33^{N}	0.142	0.094	0.232	0.032	0.02	0.386	0.238	0.566	0.087	0.35^{N}	73.1	69.6	77.8	1.9	0.54^{N}
Fraxinus ornus	29	0.262	0.111	0.411	0.072	0.78^{N}	0.083	0.031	0.141	0.026	0.73^{N}	0.179	0.073	0.271	0.049	0.52^{N}	68.4	63.4	78.4	3.9	0.02
Lamium album	35	0.139	0.089	0.343	0.047	0.00	0.036	0.023	0.081	0.010	0.00	0.103	0.061	0.262	0.038	0.00	73.5	60.4	79.7	3.9	0.01
Lupinus polyphyllus	37	0.287	0.137	0.406	0.080	0.02	0.049	0.023	0.071	0.014	0.10^{N}	0.238	0.114	0.335	0.067	0.02	82.8	80.5	84.8	1.1	0.32^{N}
Oemleria cerasiformis	32	0.345	0.226	0.474	0.066	0.62^{N}	0.106	0.073	0.180	0.021	0.02	0.238	0.153	0.344	0.051	0.26^{N}	68.9	61.9	75.5	3.5	0.56^{N}
Plantago lanceolata	29	0.692	0.070	1.484	0.297	0.05	0.086	0.013	0.130	0.026	0.46^{N}	0.606	0.057	1.365	0.284	0.04	86.3	80.0	92.6	4.6	0.00
Plantago major	28	0.771	0.376	1.386	0.267	0.23^{N}	0.165	0.080	0.317	0.059	0.24^{N}	0.606	0.296	1.069	0.210	0.22^{N}	78.6	75.7	81.5	1.5	0.66^{N}
Robinia pseudoacacia	31	0.089	0.046	0.146	0.028	0.25^{N}	0.031	0.012	0.053	0.012	0.05	0.058	0.033	0.093	0.017	0.31^{N}	65.7	60.6	74.6	4.4	0.00
Rosa arvensis - shady	33	0.035	0.022	0.077	0.011	0.00	0.017	0.010	0.040	0.006	0.00	0.018	0.011	0.037	0.006	0.00	51.9	45.2	60.0	4.2	0.32^{N}
Rosa arvensis - sunny	29	0.061	0.034	0.100	0.017	0.26^{N}	0.030	0.017	0.051	0.009	0.09^{N}	0.032	0.017	0.050	0.008	0.67^{N}	52.0	44.4	57.8	3.1	0.81^{N}
Salix pentandra	28	0.450	0.146	0.915	0.212	0.12^{N}	0.151	0.049	0.322	0.072	0.18^{N}	0.299	0.097	0.593	0.145	0.06^{N}	66.2	60.6	79.3	4.1	0.02
Secale cereale	30	0.089	0.030	0.311	0.058	0.00	0.025	0.009	0.084	0.015	0.00	0.065	0.020	0.227	0.043	0.00	72.0	65.8	77.1	3.1	0.50^{N}
Sorbus aucuparia	34	0.057	0.031	0.117	0.017	0.00	0.023	0.012	0.046	0.007	0.02	0.034	0.019	0.071	0.010	0.00	59.4	56.8	62.1	1.5	0.36^{N}
Syringa ×chinensis	38	0.184	0.098	0.308	0.062	0.04	0.067	0.032	0.117	0.024	0.06^{N}	0.117	0.066	0.191	0.038	0.02	63.9	59.1	67.3	2.0	0.14^{N}
Syringa ×prestoniae	37	1.030	0.445	1.924	0.354	0.31^{N}	0.244	0.110	0.412	0.075	0.25^{N}	0.787	0.334	1.670	0.293	0.14^{N}	75.8	70.3	86.8	3.3	0.08^{N}
Syringa josikaea	30	0.683	0.184	1.498	0.382	0.02	0.184	0.080	0.359	0.074	0.04	0.500	0.098	1.167	0.316	0.02	69.6	51.9	81.5	9.3	0.00
Syringa meyeri	35	0.155	0.095	0.241	0.036	0.29^{N}	0.056	0.028	0.093	0.014	0.57^{N}	0.100	0.060	0.149	0.025	0.22^{N}	64.1	51.9	74.8	4.4	0.02
Syringa vulgaris	32	0.793	0.522	1.071	0.150	0.35^{N}	0.270	0.168	0.377	0.058	0.04	0.523	0.354	0.730	0.102	0.28^{N}	66.0	53.1	69.9	3.5	0.00
Trifolium repens	36	0.016	0.006	0.031	0.006	0.37^{N}	0.004	0.002	0.007	0.001	0.04	0.012	0.004	0.024	0.005	0.43^{N}	73.7	57.1	87.5	5.7	0.08^{N}
Vinca minor - current year	39	0.077	0.047	0.122	0.021	0.08^{N}	0.017	0.009	0.029	0.005	0.29^{N}	0.060	0.034	0.093	0.017	0.16^{N}	77.4	70.8	90.5	3.9	0.02
Vinca minor - previous year	31	0.126	0.079	0.213	0.031	0.07^{N}	0.045	0.026	0.073	0.014	0.03	0.081	0.048	0.146	0.020	0.04	64.2	49.6	75.7	5.0	0.20^{N}
Wisteria floribunda	35	0.197	0.046	0.337	0.068	0.76^{N}	0.047	0.013	0.083	0.018	0.71^{N}	0.150	0.033	0.259	0.051	0.62^{N}	76.3	66.0	79.7	2.7	0.00
