# Supplementary material for: Is Shape of a Fresh and Dried Leaf the Same?
Source: PLoS One. 2016 Apr 5;11(4):e0153071. doi: 10.1371/journal.pone.0153071 (PMC4821626; doi:10.1371/journal.pone.0153071)
Supplement: S3 Table — SD = standard deviation; SW p = p-value in Shapiro-Wilk test, where N indicates normal distribution. (PDF) [file pone.0153071.s004.pdf]

Table S3. Basic statistics on principal component 1 (PC1) of analysed leaves/leaflets (SD = standard deviation; SW p = p-value in Shapiro-Wilk test, where ^{N} indicates normal distribution).

		**PC1 (fresh)**					**PC1 (dried)**					**ΔPC1**				
**Group**	**N**	**Mean**	**Min**	**Max**	**SD**	**SW p**	**Mean**	**Min**	**Max**	**SD**	**SW p**	**Mean**	**Min**	**Max**	**SD**	**SW p**
All samples	794	0.0036	-0.4196	0.3965	0.1637	0.00	-0.0036	-0.4269	0.3769	0.1630	0.00	0.0072	-0.0134	0.0376	0.0077	0.00
Betula pendula	36	0.1324	0.0456	0.2191	0.0427	0.79^{N}	0.1232	0.0334	0.2085	0.0421	0.84^{N}	0.0093	0.0041	0.0141	0.0027	0.37^{N}
Fagus sylvatica	34	0.1805	0.1312	0.2221	0.0284	0.07^{N}	0.1777	0.1230	0.2222	0.0293	0.14^{N}	0.0028	-0.0047	0.0114	0.0044	0.21^{N}
Ficus retusa	36	-0.0343	-0.1158	0.0597	0.0429	0.38^{N}	-0.0542	-0.1414	0.0426	0.0433	0.51^{N}	0.0199	0.0070	0.0344	0.0068	0.80^{N}
Fraxinus ornus	29	0.0452	-0.1092	0.1790	0.0748	0.05	0.0415	-0.1168	0.1702	0.0759	0.03	0.0037	-0.0069	0.0127	0.0045	0.95^{N}
Lamium album	35	-0.0458	-0.0996	0.0634	0.0395	0.02	-0.0544	-0.1070	0.0529	0.0389	0.02	0.0087	0.0009	0.0162	0.0036	0.77^{N}
Lupinus polyphyllus	37	-0.1954	-0.2450	-0.1547	0.0261	0.06^{N}	-0.2004	-0.2525	-0.1580	0.0267	0.11^{N}	0.0050	0.0004	0.0102	0.0026	0.45^{N}
Oemleria cerasiformis	32	-0.0934	-0.1873	0.0046	0.0496	0.60^{N}	-0.0959	-0.1896	-0.0046	0.0491	0.54^{N}	0.0025	-0.0012	0.0113	0.0027	0.00
Plantago lanceolata	29	-0.3189	-0.3814	-0.2407	0.0270	0.10^{N}	-0.3238	-0.3878	-0.2461	0.0267	0.14^{N}	0.0049	-0.0007	0.0085	0.0022	0.58^{N}
Plantago major	28	0.1838	0.1243	0.2877	0.0398	0.16^{N}	0.1794	0.1171	0.2798	0.0401	0.13^{N}	0.0044	-0.0041	0.0117	0.0041	0.52^{N}
Robinia pseudoacacia	31	0.2611	0.1047	0.3965	0.0588	0.70^{N}	0.2428	0.0783	0.3769	0.0599	0.54^{N}	0.0182	0.0016	0.0376	0.0095	0.49^{N}
Rosa arvensis - shady	33	0.1262	0.0217	0.2401	0.0573	0.63^{N}	0.1225	0.0122	0.2320	0.0605	0.50^{N}	0.0037	-0.0117	0.0210	0.0082	0.76^{N}
Rosa arvensis - sunny	29	0.1343	0.0335	0.2180	0.0478	0.70^{N}	0.1292	0.0242	0.2149	0.0497	0.73^{N}	0.0051	-0.0100	0.0145	0.0067	0.09^{N}
Salix pentandra	28	-0.0688	-0.2222	0.0903	0.0688	0.98^{N}	-0.0731	-0.2304	0.0799	0.0694	0.99^{N}	0.0043	-0.0013	0.0112	0.0032	0.64^{N}
Secale cereale	30	-0.3979	-0.4196	-0.3628	0.0145	0.01	-0.4062	-0.4269	-0.3735	0.0133	0.01	0.0082	0.0044	0.0171	0.0032	0.01
Sorbus aucuparia	34	0.0344	-0.0469	0.2384	0.0525	0.00	0.0157	-0.0523	0.2165	0.0510	0.00	0.0186	0.0054	0.0305	0.0063	0.84^{N}
Syringa ×chinensis	38	-0.0372	-0.0761	0.0119	0.0210	0.39^{N}	-0.0417	-0.0810	0.0070	0.0217	0.42^{N}	0.0046	-0.0012	0.0088	0.0028	0.10^{N}
Syringa ×prestoniae	37	0.0269	-0.0629	0.1108	0.0354	0.57^{N}	0.0196	-0.0757	0.1055	0.0365	0.81^{N}	0.0074	-0.0033	0.0181	0.0045	0.98^{N}
Syringa josikaea	30	-0.0709	-0.1978	0.0301	0.0635	0.11^{N}	-0.0763	-0.2082	0.0204	0.0646	0.17^{N}	0.0054	-0.0045	0.0156	0.0044	0.24^{N}
Syringa meyeri	35	0.1445	-0.0221	0.3145	0.0949	0.15^{N}	0.1336	-0.0298	0.2940	0.0952	0.11^{N}	0.0110	-0.0119	0.0301	0.0086	0.84^{N}
Syringa vulgaris	32	0.1248	0.0513	0.1567	0.0268	0.01	0.1250	0.0541	0.1587	0.0270	0.01	-0.0002	-0.0065	0.0067	0.0029	0.99^{N}
Trifolium repens	36	0.2002	0.0952	0.3190	0.0492	0.40^{N}	0.1903	0.0665	0.3214	0.0524	0.29^{N}	0.0099	-0.0134	0.0344	0.0120	0.65^{N}
Vinca minor - current year	39	-0.0894	-0.1593	0.0278	0.0356	0.04	-0.0910	-0.1611	0.0240	0.0359	0.04	0.0016	-0.0043	0.0065	0.0027	0.55^{N}
Vinca minor - previous year	31	-0.0881	-0.1573	0.1884	0.0868	0.00	-0.0893	-0.1604	0.1906	0.0877	0.00	0.0012	-0.0047	0.0068	0.0026	0.39^{N}
Wisteria floribunda	35	-0.0899	-0.1694	-0.0398	0.0327	0.19^{N}	-0.1002	-0.1717	-0.0472	0.0304	0.46^{N}	0.0102	-0.0042	0.0203	0.0059	0.41^{N}
