# Supplementary material for: Is Shape of a Fresh and Dried Leaf the Same?
Source: PLoS One. 2016 Apr 5;11(4):e0153071. doi: 10.1371/journal.pone.0153071 (PMC4821626; doi:10.1371/journal.pone.0153071)
Supplement: S4 Table — SD = standard deviation; SW p = p-value in Shapiro-Wilk test, where N indicates normal distribution. (PDF) [file pone.0153071.s005.pdf]

Table S4. Basic statistics on principal component 2 (PC2) of analysed leaves/leaflets (SD = standard deviation; SW p = p-value in Shapiro-Wilk test, where ^{N} indicates normal distribution).

		PC2 (fresh)					PC2 (dried)					ΔPC2				
**Group**	**N**	**Mean**	**Min**	**Max**	**SD**	**SW p**	**Mean**	**Min**	**Max**	**SD**	**SW p**	**Mean**	**Min**	**Max**	**SD**	**SW p**
All samples	794	0.0001	-0.1719	0.1200	0.0607	0.00	-0.0001	-0.1821	0.1255	0.0625	0.00	0.0002	-0.0171	0.0208	0.0057	0.00
Betula pendula	36	-0.1512	-0.1719	-0.1261	0.0107	0.52^{N}	-0.1525	-0.1728	-0.1225	0.0116	0.48^{N}	0.0013	-0.0044	0.0084	0.0030	0.79^{N}
Fagus sylvatica	34	0.0367	0.0135	0.0600	0.0121	0.55^{N}	0.0364	0.0113	0.0615	0.0127	0.51^{N}	0.0003	-0.0039	0.0043	0.0019	0.97^{N}
Ficus retusa	36	0.0439	0.0144	0.0814	0.0153	0.63^{N}	0.0521	0.0206	0.0934	0.0155	0.28^{N}	-0.0082	-0.0150	-0.0020	0.0035	0.47^{N}
Fraxinus ornus	29	0.0207	-0.0154	0.0773	0.0261	0.05	0.0237	-0.0126	0.0815	0.0272	0.01	-0.0030	-0.0089	0.0032	0.0027	0.89^{N}
Lamium album	35	-0.1432	-0.1683	-0.1211	0.0114	0.19^{N}	-0.1543	-0.1821	-0.1296	0.0125	0.20^{N}	0.0111	0.0031	0.0208	0.0043	0.73^{N}
Lupinus polyphyllus	37	-0.0056	-0.0168	0.0040	0.0055	0.63^{N}	-0.0067	-0.0173	0.0046	0.0054	0.77^{N}	0.0011	-0.0030	0.0071	0.0022	0.48^{N}
Oemleria cerasiformis	32	0.0429	0.0141	0.0811	0.0156	0.42^{N}	0.0425	0.0139	0.0781	0.0152	0.27^{N}	0.0004	-0.0018	0.0066	0.0018	0.00
Plantago lanceolata	29	0.0273	0.0075	0.0475	0.0115	0.17^{N}	0.0285	0.0082	0.0493	0.0116	0.34^{N}	-0.0012	-0.0051	0.0036	0.0019	0.52^{N}
Plantago major	28	0.0117	-0.0153	0.0382	0.0141	0.71^{N}	0.0120	-0.0195	0.0374	0.0148	0.44^{N}	-0.0003	-0.0041	0.0042	0.0022	0.65^{N}
Robinia pseudoacacia	31	0.0074	-0.0436	0.0450	0.0207	0.11^{N}	-0.0038	-0.0577	0.0426	0.0214	0.23^{N}	0.0112	0.0025	0.0196	0.0048	0.22^{N}
Rosa arvensis - shady	33	0.0707	0.0487	0.0955	0.0122	0.78^{N}	0.0684	0.0475	0.0957	0.0126	0.49^{N}	0.0023	-0.0103	0.0124	0.0051	0.95^{N}
Rosa arvensis - sunny	29	0.0644	0.0283	0.0985	0.0219	0.06^{N}	0.0619	0.0224	0.0933	0.0230	0.04	0.0026	-0.0098	0.0180	0.0066	0.63^{N}
Salix pentandra	28	-0.0259	-0.0513	0.0134	0.0147	0.00	-0.0264	-0.0467	0.0142	0.0147	0.00	0.0005	-0.0046	0.0038	0.0021	0.17^{N}
Secale cereale	30	0.0072	-0.0004	0.0138	0.0041	0.13^{N}	0.0093	0.0009	0.0158	0.0042	0.13^{N}	-0.0021	-0.0063	0.0024	0.0018	0.99^{N}
Sorbus aucuparia	34	0.0279	-0.0116	0.0647	0.0171	0.69^{N}	0.0205	-0.0227	0.0648	0.0178	0.87^{N}	0.0074	-0.0001	0.0148	0.0035	0.78^{N}
Syringa ×chinensis	38	-0.0549	-0.0755	-0.0233	0.0112	0.27^{N}	-0.0536	-0.0732	-0.0233	0.0108	0.47^{N}	-0.0013	-0.0042	0.0021	0.0014	0.90^{N}
Syringa ×prestoniae	37	-0.0187	-0.0423	0.0153	0.0130	0.56^{N}	-0.0168	-0.0404	0.0242	0.0147	0.11^{N}	-0.0019	-0.0090	0.0028	0.0030	0.30^{N}
Syringa josikaea	30	0.0344	-0.0767	0.1200	0.0457	0.11^{N}	0.0368	-0.0766	0.1255	0.0470	0.10^{N}	-0.0024	-0.0132	0.0048	0.0043	0.01
Syringa meyeri	35	0.0251	-0.0266	0.0862	0.0300	0.26^{N}	0.0303	-0.0217	0.0926	0.0308	0.17^{N}	-0.0052	-0.0171	0.0019	0.0045	0.28^{N}
Syringa vulgaris	32	-0.0930	-0.1063	-0.0697	0.0102	0.03	-0.0948	-0.1080	-0.0699	0.0109	0.01	0.0018	-0.0034	0.0056	0.0024	0.39^{N}
Trifolium repens	36	0.0541	0.0287	0.0734	0.0086	0.20^{N}	0.0560	0.0274	0.0751	0.0100	0.16^{N}	-0.0019	-0.0146	0.0160	0.0076	0.57^{N}
Vinca minor - current year	39	0.0145	-0.0126	0.0300	0.0092	0.09^{N}	0.0148	-0.0134	0.0309	0.0095	0.06^{N}	-0.0002	-0.0033	0.0039	0.0016	0.81^{N}
Vinca minor - previous year	31	0.0143	-0.0083	0.0379	0.0112	0.97^{N}	0.0153	-0.0073	0.0333	0.0104	0.72^{N}	-0.0009	-0.0058	0.0153	0.0035	0.00
Wisteria floribunda	35	0.0240	-0.0132	0.0604	0.0150	0.84^{N}	0.0293	-0.0078	0.0631	0.0143	0.84^{N}	-0.0054	-0.0123	0.0024	0.0031	0.97^{N}
