# Supplementary material for: Is Shape of a Fresh and Dried Leaf the Same?
Source: PLoS One. 2016 Apr 5;11(4):e0153071. doi: 10.1371/journal.pone.0153071 (PMC4821626; doi:10.1371/journal.pone.0153071)
Supplement: S5 Table — SD = standard deviation; SW p = p-value in Shapiro-Wilk test, where N indicates normal distribution. (PDF) [file pone.0153071.s006.pdf]

Table S5. Basic statistics on sin α and cos α (SD = standard deviation; SW p = p-value in Shapiro-Wilk test, where [N] indicates normal distribution).

		sin α					cos α				
**Group**	**N**	**Mean**	**Min**	**Max**	**SD**	**SW p**	**Mean**	**Min**	**Max**	**SD**	**SW p**
All samples	794	0.644	-1.000	1.000	0.538	0.00	-0.024	-1.000	1.000	0.544	0.00
Betula pendula	36	0.932	0.544	1.000	0.093	0.00	0.151	-0.600	0.839	0.321	1.00[N]
Fagus sylvatica	34	0.406	-0.994	1.000	0.737	0.00	0.083	-0.996	0.995	0.557	0.46[N]
Ficus retusa	36	0.883	0.502	0.998	0.123	0.00	-0.401	-0.865	-0.067	0.213	0.13[N]
Fraxinus ornus	29	0.526	-0.928	1.000	0.567	0.00	-0.513	-1.000	0.676	0.394	0.03
Lamium album	35	0.588	0.189	0.970	0.188	0.36[N]	0.765	0.243	0.982	0.190	0.00
Lupinus polyphyllus	37	0.895	0.341	1.000	0.134	0.00	0.164	-0.738	0.940	0.398	0.72[N]
Oemleria cerasiformis	32	0.717	-0.743	1.000	0.426	0.00	0.084	-0.970	0.999	0.559	0.24[N]
Plantago lanceolata	29	0.874	-0.415	0.998	0.267	0.00	-0.231	-0.910	0.647	0.343	0.94[N]
Plantago major	28	0.608	-0.970	1.000	0.650	0.00	-0.007	-0.806	0.995	0.481	0.43[N]
Robinia pseudoacacia	31	0.783	0.115	0.988	0.208	0.00	0.544	0.154	0.993	0.226	0.67[N]
Rosa arvensis - shady	33	0.229	-0.991	1.000	0.798	0.00	0.209	-0.997	0.956	0.543	0.03
Rosa arvensis - sunny	29	0.482	-1.000	1.000	0.626	0.00	0.172	-0.993	0.991	0.611	0.08[N]
Salix pentandra	28	0.739	-0.829	0.993	0.434	0.00	0.034	-0.997	0.959	0.530	0.27[N]
Secale cereale	30	0.942	0.643	1.000	0.070	0.00	-0.240	-0.765	0.464	0.227	0.13[N]
Sorbus aucuparia	34	0.903	0.463	1.000	0.099	0.00	0.381	-0.006	0.886	0.175	0.36[N]
Syringa ×chinensis	38	0.745	-0.974	1.000	0.488	0.00	-0.259	-0.972	0.683	0.387	0.09[N]
Syringa ×prestoniae	37	0.835	-0.565	1.000	0.306	0.00	-0.221	-0.999	0.520	0.409	0.60[N]
Syringa josikaea	30	0.775	-0.382	1.000	0.400	0.00	-0.248	-0.982	0.664	0.436	0.70[N]
Syringa meyeri	35	0.750	-0.679	1.000	0.393	0.00	-0.400	-0.999	0.248	0.363	0.16[N]
Syringa vulgaris	32	-0.001	-0.994	0.998	0.710	0.00	0.442	-0.970	0.999	0.571	0.00
Trifolium repens	36	0.476	-0.999	1.000	0.718	0.00	-0.121	-0.995	0.880	0.515	0.30[N]
Vinca minor - current year	39	0.317	-0.999	1.000	0.727	0.00	-0.078	-0.999	1.000	0.623	0.06[N]
Vinca minor - previous year	31	0.259	-0.992	1.000	0.657	0.00	-0.408	-0.999	1.000	0.600	0.00
Wisteria floribunda	35	0.759	-0.394	1.000	0.342	0.00	-0.469	-0.992	0.116	0.305	0.04
