# Supplementary material for: Is Shape of a Fresh and Dried Leaf the Same?
Source: PLoS One. 2016 Apr 5;11(4):e0153071. doi: 10.1371/journal.pone.0153071 (PMC4821626; doi:10.1371/journal.pone.0153071)
Supplement: S6 Table — SD = standard deviation; SW p = p-value in Shapiro-Wilk test, where N indicates normal distribution. (PDF) [file pone.0153071.s007.pdf]

Table S6. Basic statistics on shape change and specific leaf area (SLA) of analysed leaves/leaflets (SD = standard deviation; SW p = p-value in Shapiro-Wilk test, where [N] indicates normal distribution).

		Shape change (vector length)					SLA [cm²/g]				
**Group**	**N**	**Mean**	**Min**	**Max**	**SD**	**SW p**	**Mean**	**Min**	**Max**	**SD**	**SW p**
All samples	794	0.0095	0.0003	0.0403	0.0073	0.00	228	65	570	90	0.00
Betula pendula	36	0.0099	0.0047	0.0148	0.0025	0.44[N]	177	151	221	18	0.06[N]
Fagus sylvatica	34	0.0046	0.0005	0.0117	0.0031	0.02	435	381	519	32	0.47[N]
Ficus retusa	36	0.0221	0.0109	0.0346	0.0057	0.73[N]	136	94	165	12	0.17[N]
Fraxinus ornus	29	0.0064	0.0006	0.0127	0.0030	0.44[N]	199	166	297	28	0.00
Lamium album	35	0.0145	0.0048	0.0244	0.0042	0.99[N]	269	223	325	22	0.99[N]
Lupinus polyphyllus	37	0.0055	0.0005	0.0123	0.0027	0.76[N]	269	197	350	34	0.58[N]
Oemleria cerasiformis	32	0.0032	0.0004	0.0131	0.0027	0.00	263	167	384	53	0.56[N]
Plantago lanceolata	29	0.0054	0.0016	0.0098	0.0021	0.80[N]	314	192	508	102	0.01
Plantago major	28	0.0058	0.0022	0.0119	0.0026	0.16[N]	205	170	257	23	0.09[N]
Robinia pseudoacacia	31	0.0222	0.0067	0.0403	0.0088	0.64[N]	245	187	350	44	0.01
Rosa arvensis - shady	33	0.0089	0.0006	0.0231	0.0054	0.39[N]	212	154	289	30	0.60[N]
Rosa arvensis - sunny	29	0.0102	0.0043	0.0189	0.0039	0.15[N]	143	112	186	21	0.09[N]
Salix pentandra	28	0.0050	0.0004	0.0115	0.0028	0.12[N]	177	142	279	28	0.00
Secale cereale	30	0.0087	0.0044	0.0171	0.0031	0.01	270	192	337	34	0.77[N]
Sorbus aucuparia	34	0.0204	0.0072	0.0322	0.0061	0.93[N]	172	151	210	11	0.14[N]
Syringa ×chinensis	38	0.0051	0.0004	0.0093	0.0025	0.24[N]	161	124	198	21	0.10[N]
Syringa ×prestoniae	37	0.0084	0.0017	0.0183	0.0042	0.53[N]	195	139	267	33	0.11[N]
Syringa josikaea	30	0.0077	0.0008	0.0162	0.0036	0.44[N]	236	176	330	41	0.16[N]
Syringa meyeri	35	0.0141	0.0028	0.0317	0.0066	0.53[N]	151	121	243	24	0.00
Syringa vulgaris	32	0.0037	0.0006	0.0085	0.0019	0.43[N]	152	106	183	22	0.03
Trifolium repens	36	0.0153	0.0017	0.0380	0.0082	0.55[N]	333	212	570	65	0.00
Vinca minor - current year	39	0.0030	0.0003	0.0069	0.0019	0.11[N]	237	178	321	33	0.79[N]
Vinca minor - previous year	31	0.0035	0.0003	0.0153	0.0029	0.00	102	65	204	32	0.00
Wisteria floribunda	35	0.0128	0.0065	0.0204	0.0036	0.26[N]	402	253	526	53	0.73[N]
