# Supplementary material for: Is Shape of a Fresh and Dried Leaf the Same?
Source: PLoS One. 2016 Apr 5;11(4):e0153071. doi: 10.1371/journal.pone.0153071 (PMC4821626; doi:10.1371/journal.pone.0153071)
Supplement: S7 Table — Bolded values (p > 0.05) indicate that the compared populations do not differ significantly. (PDF) [file pone.0153071.s008.pdf]

Table S7. Results (p-values) of paired Student's t-test or Wilcoxon signed-rank test for repeated measurements of principal component 1 (PC1), PC2, area and mass of analysed fresh and dried leaves/leaflets. Bolded values ($p > 0.05$) indicate that the compared populations do not differ significantly.

		**PC1**		**PC2**		**Area**		**Mass**	
**Group**	**N**	**Student's test**	**Wilcoxon test**	**Student's test**	**Wilcoxon test**	**Student's test**	**Wilcoxon test**	**Student's test**	**Wilcoxon test**
All samples	794		0.00		**0.30**		0.00		0.00
Betula pendula	36	0.00		0.02		0.00		0.00	
Fagus sylvatica	34	0.00		**0.39**		0.00		0.00	
Ficus retusa	36	0.00		0.00		0.00			0.00
Fraxinus ornus	29		0.00		0.00	0.00		0.00	
Lamium album	35		0.00	0.00			0.00		0.00
Lupinus polyphyllus	37	0.00		0.00			0.00		0.00
Oemleria cerasiformis	32	0.00		**0.18**		0.00			0.00
Plantago lanceolata	29	0.00		0.00		0.00			0.00
Plantago major	28	0.00		**0.44**		0.00		0.00	
Robinia pseudoacacia	31	0.00		0.00		0.00			0.00
Rosa arvensis - shady	33	0.01		0.01			0.00		0.00
Rosa arvensis - sunny	29	0.00			**0.06**	0.00		0.00	
Salix pentandra	28	0.00			**0.12**	0.00		0.00	
Secale cereale	30		0.00	0.00			0.00		0.00
Sorbus aucuparia	34		0.00	0.00			0.00		0.00
Syringa ×chinensis	38	0.00		0.00			0.00		0.00
Syringa ×prestoniae	37	0.00		0.00		0.00		0.00	
Syringa josikaea	30	0.00		0.00			0.00		0.00
Syringa meyeri	35	0.00		0.00		0.00		0.00	
Syringa vulgaris	32		**0.68**		0.00	0.00			0.00
Trifolium repens	36	0.00		**0.15**		0.00			0.00
Vinca minor - current year	39		0.00	**0.37**			0.00	0.00	
Vinca minor - previous year	31		0.01	**0.14**		0.00			0.00
Wisteria floribunda	35	0.00		0.00		0.00			0.00
