# Supplementary material for: Is Shape of a Fresh and Dried Leaf the Same?
Source: PLoS One. 2016 Apr 5;11(4):e0153071. doi: 10.1371/journal.pone.0153071 (PMC4821626; doi:10.1371/journal.pone.0153071)
Supplement: S8 Table — Bolded r values are significant with p < 0.05. (PDF) [file pone.0153071.s009.pdf]

Table S8. Correlations between shape change (vector length) and selected variables expressed as coefficient r values. Bolded r values are significant with $p < 0.05$.

Variable	Group	Mass (fresh)	Mass (dried)	Area (fresh)	Area (dried)	SLA	Mass loss	Area loss	Area loss [relative]	Mass loss [relative]
Shape change (vector length)	All samples	**-0.22**	**-0.22**	**-0.28**	**-0.30**	-0.04	**-0.21**	-0.02	**0.64**	**-0.12**
	Betula pendula	-0.05	-0.06	-0.05	-0.05	0.12	-0.05	-0.02	0.13	0.04
	Fagus sylvatica	-0.21	-0.21	-0.22	-0.24	0.05	-0.21	0.34	**0.61**	-0.01
	Ficus retusa	-0.19	-0.11	-0.28	-0.29	**-0.37**	-0.21	-0.17	0.19	-0.22
	Fraxinus ornus	**-0.65**	**-0.67**	**-0.63**	**-0.64**	0.25	**-0.60**	-0.21	**0.56**	0.27
	Lamium album	-0.02	-0.11	-0.07	-0.09	0.19	0.01	**0.35**	**0.48**	0.19
	Lupinus polyphyllus	**-0.58**	**-0.50**	**-0.57**	**-0.61**	0.10	**-0.59**	**0.35**	**0.82**	-0.21
	Oemleria cerasiformis	-0.24	-0.25	-0.21	-0.22	-0.04	-0.22	0.01	0.21	-0.01
	Plantago lanceolata	0.19	0.02	0.15	0.09	0.09	0.19	**0.57**	**0.80**	0.08
	Plantago major	-0.32	-0.31	**-0.37**	**-0.39**	-0.02	-0.32	-0.20	**0.46**	0.01
	Robinia pseudoacacia	0.05	0.10	-0.03	-0.07	-0.23	0.02	**0.41**	**0.68**	-0.15
	Rosa arvensis - shady	-0.23	-0.32	-0.17	-0.18	0.34	-0.11	-0.06	0.23	**0.43**
	Rosa arvensis - sunny	0.14	0.10	0.14	0.13	0.09	0.17	0.30	0.10	0.14
	Salix pentandra	-0.15	-0.23	-0.19	-0.21	0.34	-0.11	0.04	**0.71**	0.27
	Secale cereale	-0.34	-0.35	-0.35	**-0.39**	0.23	-0.33	-0.00	**0.86**	-0.09
	Sorbus aucuparia	0.15	0.12	0.07	0.02	-0.18	0.16	**0.40**	**0.59**	0.18
	Syringa ×chinensis	**-0.33**	-0.29	**-0.40**	**-0.42**	-0.01	**-0.36**	-0.02	**0.63**	-0.06
	Syringa ×prestoniae	-0.18	-0.32	-0.23	-0.28	0.11	-0.14	0.18	**0.67**	0.15
	Syringa josikaea	-0.04	-0.03	-0.10	-0.12	-0.16	-0.04	0.07	0.32	-0.01
	Syringa meyeri	-0.29	-0.33	-0.24	-0.28	0.33	-0.25	0.17	**0.53**	0.16
	Syringa vulgaris	0.21	0.17	0.20	0.19	-0.02	0.20	**0.41**	0.26	-0.02
	Trifolium repens	**-0.44**	**-0.42**	**-0.47**	**-0.49**	0.05	**-0.42**	-0.01	**0.45**	-0.16
	Vinca minor - current year	0.26	0.02	0.16	0.13	0.21	0.32	**0.46**	**0.50**	**0.37**
	Vinca minor - previous	-0.04	-0.11	0.08	0.07	0.18	0.02	0.32	**0.41**	0.20
	Wisteria floribunda	**0.51**	**0.54**	**0.49**	**0.46**	**-0.38**	**0.49**	**0.65**	**0.67**	-0.22
